# Silk fibroin-derived polypeptides additives to promote hydroxyapatite nucleation in dense collagen hydrogels

**DOI:** 10.1371/journal.pone.0219429

**Published:** 2019-07-15

**Authors:** Imran Deen, Federico Rosei

**Affiliations:** Centre Énergie, Matériaux et Télécommunications, Institut national de la recherche scientifique, Varennes, QC, Canada; Brandeis University, UNITED STATES

## Abstract

Silk fibroin-derived polypeptides (FDPs) are polypeptides resulting from the enzymatic separation of the hydrophobic crystalline (C_p_) and hydrophilic electronegative amorphous (C_s_) components of silk fibroin (SF). The role of these polypeptides in promoting the nucleation of hydroxyapatite (HA) has been previously investigated, yet is still not fully understood. Here we study the potential of HA mineralization via FDPs incorporated at 1:10, 1:2 and 1:1 in a plastically compressed (PC) and dense collagen (DC) scaffold. Scaffolds were immersed in simulated body fluid (SBF) at physiological conditions (pH = 7.4, 37°C) to promote biomineralization. The effect of C_s_ and C_p_ to promote HA nucleation was investigated at different time points, and compared to pure DC scaffolds. Characterization of C_s_ and C_p_ fragments using Liquid Chromatography–Mass Spectrometry (LCMS) showed little difference in the amino acid composition of the FDPs. Results obtained *in vitro* using Attenuated Total Reflectance Fourier Transform Infrared Spectroscopy (ATR-FTIR), Scanning Electron Microscopy (SEM) X-Ray Diffraction (XRD) and mass analysis showed little difference between scaffolds that incorporated C_s_, C_p_, and DC hydrogels. These results demonstrated that silk FDPs incorporation are not yet suitable to promote HA nucleation *in vivo* without further refining the collagen-FDP system.

## Introduction

Native bone tissue is composed of organic collagen fibrils and inorganic hydroxyapatite (HA) crystals arranged in a strict hierarchical structure. The collagen fibrils provide a template for HA, with crystals nucleating and growing within the gaps of collagen [1,2]. While the nucleation of HA crystals occurs within collagen fibrils, collagen itself does not take an active role in HA nucleation, and only a small amount of apatite will form after a long period of time [3,4]. Instead, it is the non-collagenous proteins (NCPs) that are responsible for HA formation [5]. In the literature, it is demonstrated that NCPs having calcium binding and HA nucleating properties contain glutamic acid [6–8]. Glutamic acid has the ability to bind Ca^2+^ ions to negatively charged carboxylate groups, which in turn attract PO_4_^3-^ ions and increase the local concentration past the point of supersaturation [9], with a small amount (4–8 wt% [10]) of carbonate ions remaining within the HA crystal. This supersaturation reaches a critical level, and leads to nucleation sites for HA crystals [8,11,12]. However, without the NCPs, the mineralization of collagen generally does not proceed [1]. As a consequence, collagen scaffolds used in bone implants without mineralization require more than 2–3 weeks for osteointegration [13]. Currently, an increasing number of bioactive materials are being developed to replace NCPs and promote the nucleation of HA and mineralization of collagen for bone defects repair [2,13–15].

Silk is a natural protein fiber that is used by certain insects such as spiders and silkworms (*Bombyx mori*), with the former using it to make webs and the latter to form cocoons. Though spider silk is known for its outstanding mechanical properties [16], *Bombyx mori* silk from domesticated silkworms has the advantage of being available in higher quantities [17]. The latter is composed of two main proteins, sericin and fibroin. The silk fibroin (SF) is the core structural component of silk, which exhibits high tensile strength [18], while the sericin surrounds the fibroin and acts as a glue, or “gum” [19]. Due to its ease of processing, biodegradability and high tensile strength, silk fibroin is especially attractive for biomedical applications [20–22]. In particular, due to the high concentration of glutamic acid in SF [23], it has been proposed that nucleating HA on SF is possible *in vitro*, and is considered as a promising material for repairing bone defects [14,24,25].

As SF is composed of peptides that are hydrophilic (C_s_) and hydrophobic (C_p_), it is fairly straightforward to separate SF into its constituents by initially degumming (removing the sericin/silk gum by cleaving its peptide bonds) the *Bombyx mori* silk and then hydrolyzing the SF [26]. Then, by dissolving the SF with α-chymotrypsin (a digestive enzyme that breaks down proteins into polypeptides), and centrifuging the resultant, the supernatant, C_s_, and precipitate, C_p_ can be separated [27]. Once separated, it has been shown that C_s_ and C_p_ have different compositions, with C_s_ having more anionic amino acids than C_p_ [23]. Furthermore, the C_s_ component of SF has excellent biomineralization properties *in vitro*. DC hydrogels that incorporate C_s_ have been shown to form HA under physiological temperature and pH, when immersed in simulated body fluid (SBF), unlike C_p_-containing DC gels, while C_p_ showed almost no mineralization [27]. It is thought that the high concentration of glutamic acid (glu) in C_s_ [23] allows it to play a role similar to calcium-binding non-collagenous proteins (NCPs), which act as modulators for the nucleation and growth of HA nanocrystals [28–30] by attracting more Ca^2+^ ions, as well as providing more nucleation sites for HA crystals [31].

Recent research has focused on using three dimensional (3D) dense collagen scaffolds that can incorporate silk fibroin-derived polypeptides (FDPs) [27]. Previous work has shown that the compression of collagen gels [32–34] and the incorporation of FDPs have more desirable properties, such as the mineralization of hydroxyapatite (HA) [27] (uniformly, in both intra- and extrafibrillar locations [35]), increased cell proliferation and adhesion [32,33], biocompatibility with the surrounding tissue [36], reduced inflammatory response, and increased bone regeneration [37–39], and similar diffusion coefficients for oxygen and glucose to that of native collagen tissue [40,41].

While the nucleation of HA crystals occur within collagen fibrils, it is the non-collagenous proteins (NCPs) which are responsible for HA formation, and account for less than 10% of proteins found in bone. Herein, we aim at promoting the increased biomineralization of HA in DC scaffolds by incorporating FDPs. By investigating both the FDPs and the nucleation of HA in SBF [42], the potential of FDPs as biomaterials for bone grafts was also determined *in vitro*. The amino acid composition of FDPs was also assessed and compared to previous work [23]. It was hypothesized that FDP can serve as an analogue to NCPs that serves to accumulate Ca^2+^ and PO_4_^3-^ ions that form HA. While NCPs account for less than 10% of the proteins found in bone, they play an important role in bone mineralization by acting as HA nucleation centers [43]. Additionally, plastic compression (PC) removes excess water and forms a dense collagen (DC) hydrogel, in which the collagen fibril density (CFD) increases from <0.5 wt.% to approximately 8 wt.% [44]. This results in a scaffold that closely mimics collagen in native bone tissue [32,33,36,44] with a greater potential to nucleate HA. The objective was to obtain a hydrogel that has the composite structure of flexible collagen and tough HA, which gives bone its requisite strength and high fracture toughness [45–47]. Thus, in this paper we study the potential for including FDPs in DC scaffolds for bone tissue engineering (BTE).

## Methodology

### Preparation of hydrogels and bioactive additives

#### Silk fibroin polypeptides

Soluble silk fibroin-derived peptides were produced from raw *Bombyx Mori* (silkworm) silk provided by Stazione Sperimentale per la Seta (Milan, Italy). The silk sericin was degummed and the fibroins were cleaved and separated into their soluble (C_s_) and non-soluble phases (C_p_), using a method reported in the literature [27,48,49].

Silk fibroin fibers were produced through a heating cycle that removed the silk sericin. The resulting fibroin was dissolved in a concentrated aqueous solution of LiBr (Sigma) [50,51], due to LiBr being a chaotropic salt, which disrupts the bonds in protein molecules [52,53]. The dissolved fibroin results in both α-helical and β-folded structures [51], heated, then separated using dialysis to produce a 2% silk fibroin solution [48].

The resulting silk fibroin was then diluted with a 0.1 M ammonium bicarbonate solution (NH_4_CO_3_, Fisher Scientific), then enzymatically digested with α-chymotrypsin (Sigma) at an enzyme-to-substrate ratio of 1:100 to generate C_p_ and C_s_ in solution, which was incubated at 37°C for 24 hours then centrifuged to separate the supernatant (containing C_s_) from the pellet (containing C_p_). Both the supernatant and the pellet were freeze-dried to produce C_s_ and C_p_ [27].

#### Preparation of simulated body fluid

Kokubo’s SBF was created based on a protocol developed for analysing apatite formation [42]. Reagents were mixed in in 3 L of distilled water in the following order: NaCl (Fisher Scientific) (24.105 g), NaHCO_3_ (Fisher Scientific) (1.065 g), KCl (Sigma) (0.675 g), K_2_HPO_4_·3H2O (0.693 g), MgCl_2_·6H_2_O (Fisher Scientific) (0.933 g) 1.0M HCl (117 mL), CaCl_2_ (Sigma) (0.876 g), Na_2_SO_4_ (Sigma) (0.216 g), Tris(hydroxymethyl)aminomethane (Tris) (Fisher Scientific) (18.354 g), 1.0M HCl (Fisher Scientific) (0–15 mL). Tris and HCl were added concurrently to maintain a pH of ~7.4.

#### Plastically compressed dense collagen hydrogels

A solution of 13:1 10X Dulbeccos’s Modified Eagle Medium (DMEM) (Gibco) to 5N NaOH (Fisher Scientific, Sodium Hydroxide Solution 5N) was created. A 5.83 mg/mL bovine dermis (BD) collagen solution collagen solution (Devro Medical, Purified Soluble Collagen) was added at 4:1 by volume to the DMEM. The pH was adjusted to 7.4 by adding NaOH while kept at ~4 ºC using an icepack or a box filled with ice. [27,36].

To produce Dense Collagen (DC) gels, a 48-well plate (10.5 mm diameter per well) was filled with 1 mL of the above solution (DMEM, NaOH and collagen) and, similar to a previously established protocol [27], left in an incubator (Thermo Scientific, Forma Series II) at 37°C for 30 minutes. Collagen gels were then subjected to a plastic compression method [27,33,34,36] by applying 1 kPa (40 g per 350 mm^2^; 160 g for four gels) for five minutes to collagen gels placed between two nylon meshes and on top a steel mesh and blot paper (the latter to collect expelled water). The load was applied to expel water and retain collagen, and produce DC gels.

Hydrogels containing a FDP additive (C_s_ or C_p_) were created following the previously outlined protocol [27]. The process for creating DC hydrogels containing C_s_ (DC-C_s_), and C_p_ (DC-C_p_) was identical to that outlined above, with an interim step of mixing the additive in the DMEM then ultrasonicating the solution prior to adding NaOH and collagen. Collagen was added to the DMEM at 4:1, and FDPs to polymer ratios of 1:10 1:2 and 1:1 were used to determine effect of FDPs in the biomineralization of collagen gels, with pure collagen gels used as control (see Table 1). After adding collagen, the solution was magnetically stirred to ensure a homogenous solution.

**Table 1:** Solution of 10 mL collagen with additives (added prior to plastic compression).

Additive: polymer	Mass of collagen (mg)	Mass of C_s_/C_p_ (mg)	Mass of polymer and additive in solution (mg)
Collagen (ctrl)	46.8	0.0	46.8
1:10	46.8	4.7	51.5
1:2	46.8	23.4	70.2
1:1	46.8	46.8	93.6

### Mineralization of hydroxyapatite within additive-incorporated hydrogels

Both C_s_ and C_p_ were immersed in SBF at a 1:3 ratio (3 mg/mL) and placed in an incubator for 24 hours before being removed and analysed. The resulting gels were immersed in SBF (pH 7.4, 37°C) for up to two weeks, using a hydrogel to SBF ratio of 1:3 (mg/mL). The solution was replaced at two or three-day intervals by fresh SBF. Samples were taken at days 0, 3, 7, 10 and 14. The results were compared to gels that had no additives incorporated. HA nucleation in bone occurs within the gaps of collagen fibrils [48], though for scaffolds constructed *in vitro*, a high concentration of carboxyl groups was shown to lead to HA nucleation. Charged amino acids act as nucleation sites, where calcium ions are gathered through electrostatic attraction, which then attract phosphate ions until a critical concentration is reached, leading to HA formation [49–52]. The nucleation of HA is thus expected to occur around the charged Cs particles within the collagen scaffold.

### Liquid chromatography–mass spectrometry

Liquid Chromatography–Mass Spectrometry (LCMS) was carried to determine the amino acid composition of the silk fibroin derived polypeptides on a QTOF (Agilent). LCMS was carried out at IRIC-Université de Montréal Proteomics facilities. The results were analysed in Microsoft Excel.

### Particle size analysis

Particle Size Analysis (PSA) (Horiba LA-920) was performed on C_s_ and C_p_ to determine the size distribution. Isopropanol was used to create a solution that was then ultrasonicated before analysis. A refractive index of n_D_^22^ 1.55 (n_D_^22^ 1.13 in isopropanol) was used to calculate the size distribution based on values obtained from the literature [54,55].

### Fourier transform infrared spectroscopy

Attenuated Total Reflectance Fourier Transform Infrared Spectroscopy (ATR-FTIR) (Perkin Elmer, Spectrum 400) was used to characterize the mineralization that occurred in the sample, as well as any changes in the chemical composition of the sample itself. The bands associated with PO_4_^3-^, indicative of the presence of HA, were compared between timepoints and samples to monitor HA mineralization [27,56]. Additionally, IR is sensitive to the substitution of PO_4_^3-^ ions by CO_3_^2-^ ions, and can detect the presence of small amounts of carbonate, indicating the formation of carbonated hydroxyapatite [57].

Samples were prepared for ATR-FTIR analysis by first washing in dH_2_O to remove any excess SBF, then freezing in liquid nitrogen, then freeze-drying (lyophilizing) the sample until all the excess moisture had been removed. The sample was then analysed at a resolution of 2 cm^-1^ in the IR range of 4000 to 650 cm^-1^ at 32 scans per sample. The resulting spectra were normalized against the Amide I band found between 1800 to 1650 cm^-1^ for comparison (Spectrumsoftware, Perkin-Elmer) [36,58].

### X-Ray diffraction

X-Ray Diffraction (XRD) (Bruker D8, Bruker-AXS Corp.) was performed on C_p_ and C_s_ fragments, as well as freeze-dried hydrogels that had been immersed in SBF. Samples were compressed and taped onto glass slides to produce a flat, fixed surface, and XRD analysis was performed using a Cu-K_α_ source. XRD patterns were recorded from 3 to 104° 2 theta at 40 kV and 40 mA. Four frames of 25° were recorded for 150 seconds and then merged during data post processing. The resulting patterns were analysed (EVA 14.0.0.0, Bruker) and compared with spectra with peaks identified in the International Centre for Diffraction Data (ICDD) database.

### Scanning electron microscopy

Samples for Scanning Electron Microscopy (SEM) imaging were prepared by immersing the hydrogels in 30, 50, 70, 80, 90, 95 and 100% ethanol solutions to remove all the water [27,36]. The gels were then immersed in 1,1,1,3,3,3-hexamethyldisilazane (HMDS) and left overnight until all the HMDS had evaporated. All samples were sputter-coated with a layer of Au/Pd then analysed by SEM (FEI Inspect F-50 FE-SEM, FEI).

### Statistical analysis

The data was analyzed for statistical significance using a one-way ANOVA with a statistical significance level of 0.05. Tukey and Holm-Bonferroni methods were used for comparison (Origin Pro v9.0 software, OriginLab).

## Results

### Materials characterization

**Fig 1:**
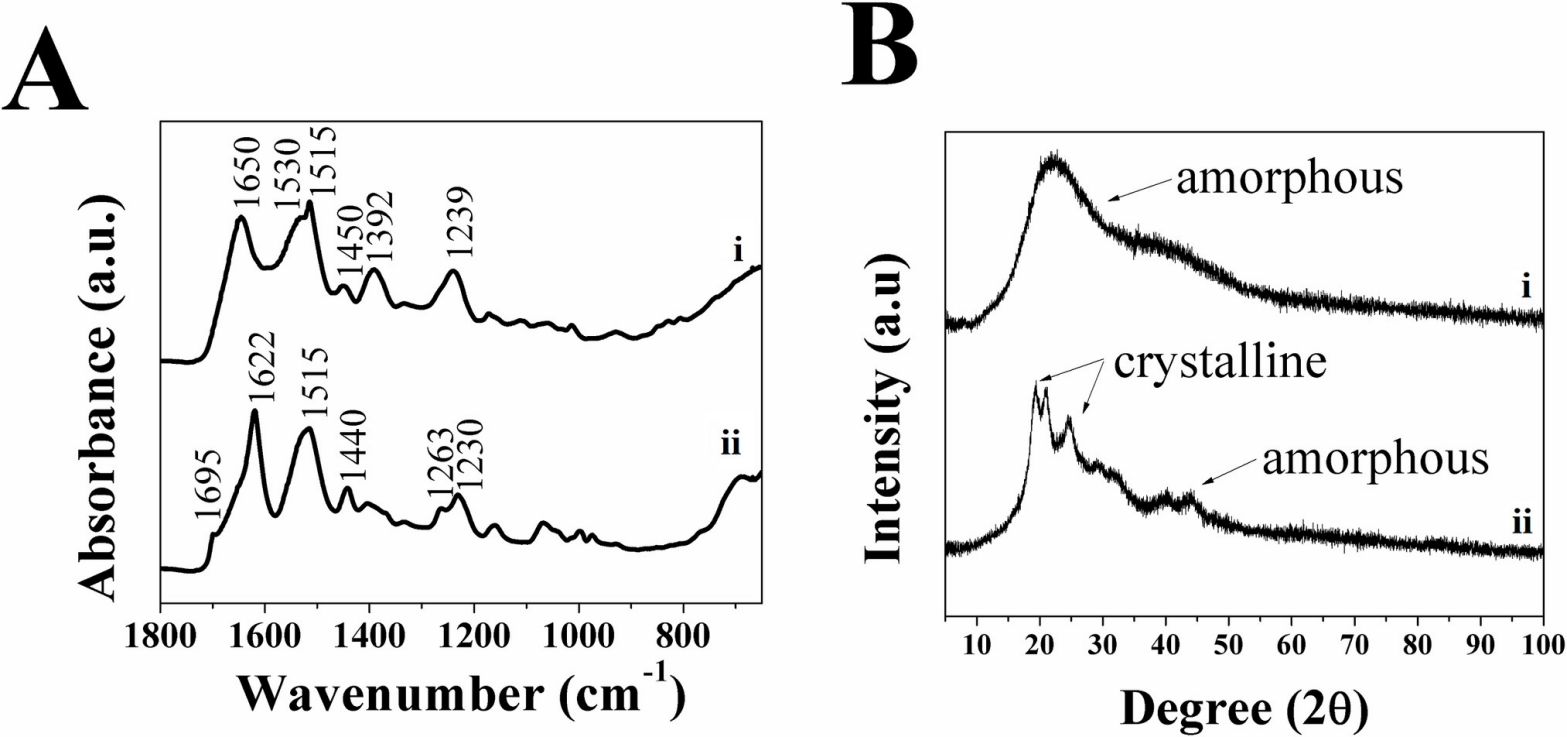
A) ATR-FTIR spectra showing secondary structures and B) XRD patterns showing amorphous/crystalline structure for as-made i) C_s_ and ii) C_p_. arrows in the XRD patterns indicate crystalline regions and amorphous regions.

Characterization of the starting materials (collagen, C_s_ and C_p_) was carried out using ATR-FTIR to determine their chemical composition, which can serve as a reference (control) for future tests. While the ATR-FTIR spectra of C_p_ and C_s_ are quite similar, still there are slight differences, such as the shoulder at 1695 cm^-1^ in C_p_ and the amide I band at 1650 cm^-1^ for C_s_. This can be attributed to C_p_ polypeptides being composed of the crystalline regions of silk fibroin and having a β-sheet type structure, while C_s_ has an α-helix type structure [16,23]. The spectra for C_p_ (see Fig 1A) exhibit a shoulder on the amide I band at 1695 cm^-1^ that is typically associated with a β-sheet structure of SF, as well as Amide I, II and III absorptions at 1622, 1515 and 1230 cm^-1^ that are characteristic of SF [27,59]. The spectra for C_s_ (see Fig 1B) differed slightly in the amide I band at 1650 cm^-1^ that is typical of an α-helix type structure, though it still have the Amide II and III bands at 1530–1515 cm^-1^ and 1239 cm^-1^, respectively [27].

**Fig 2:**
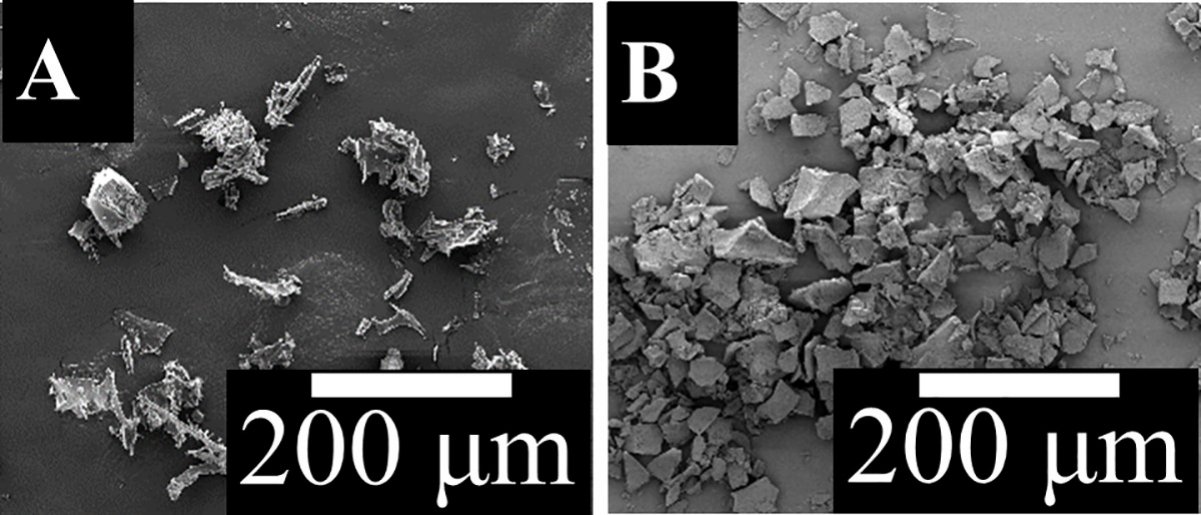
SEM images of A) C_s_ and B) C_p_ particles.

XRD patterns for solubilized C_s_ and C_p_ (see Fig 1B) fragments revealed a crystalline structure in C_p_ that is similar to XRD patterns of SF reported elsewhere [60], while C_s_ fragments resulted in spectra confirming the amorphous nature. SEM images of C_s_ and C_p_ also indicate that C_p_ has a crystalline structure, while C_s_ has an amorphous structure (see Fig 2). These results confirmed that the structure of SF is composed of both the crystalline phase of C_p_ and the amorphous phase of C_s_.

**Fig 3:**
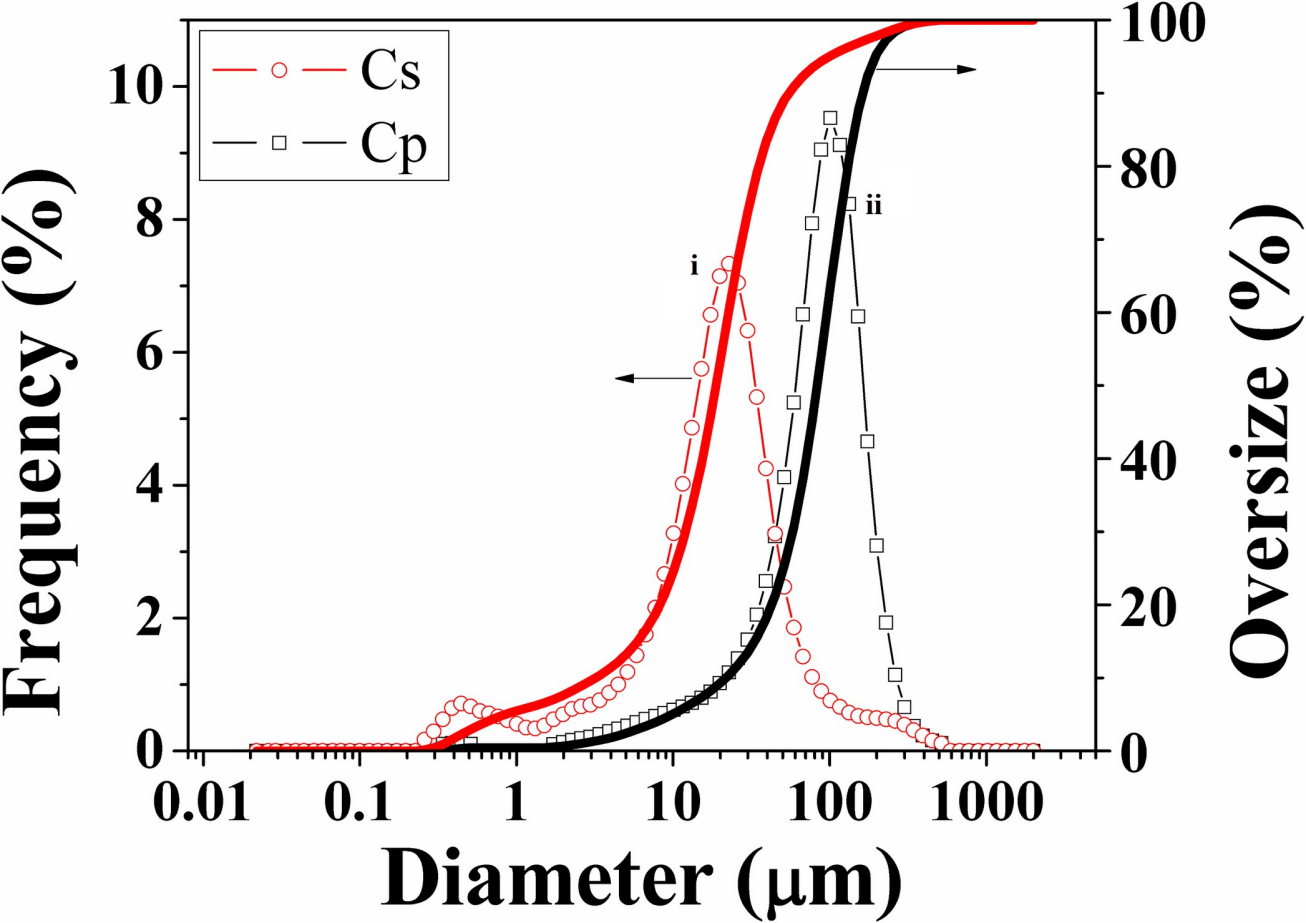
Size distribution of i) C_p_ and ii) C_s_ particles.

The size distribution of C_p_ and C_s_ particles indicates that C_p_ is significantly larger than C_s_ (see Fig 3). The mean particle size of C_p_ is 91.02 μm, while C_s_ is only 22.46 μm. The difference in size can be attributed to C_p_ having a molecular weight 4–20 times higher than C_s_ [27], but is more likely due to the structure of C_p_ being semi-crystalline, whereas C_s_ is amorphous [61–64]. The use of α-chymotrypsin to separate the two phases attacks the amorphous Cs phase, while not affecting the crystalline C_p_, resulting in larger crystalline C_p_ and smaller chunks of C_s_ [22,64,65]

LCMS results contrast with the previously assumed composition of the silk fibroin-derived polypeptides (see Table 2). LCMS showed that C_s_ and C_p_ have a similar composition to silk fibroin. The composition of C_p_ is similar to values found previously [23]. In contrast, C_s_ has a slightly more varied composition, though it has a lower concentration of sequences rich in aspartic acid and glutamic acid residues compared to similar results reported in the literature [23,55]. The values obtained from LCMS match the structure seen previously, with the Glycine amino acids alternating with other amino acids (except for one Ala-Ala link) [66]. The NCBI database for the amino acid composition of silk fibroin polypeptides that are composed of a mixture of Heavy Chains (HC) (NCBI Reference Sequence: NP_001106733.1) and Light Chains (LC) (NCBI Reference Sequence: NP_001037488.1) shows that C_s_ and C_p_ have a similar composition to silk fibroin as well, though with a lower concentration of glutamic acid (HC– 0.57%, LC– 1.91%) and aspartic acid (HC– 0.48%, LC– 6.49%).

**Table 2:** Comparison of amino acid composition for fibroin-derived polypeptides, C_p_ and C_s_, obtained from LCMS (Right–from literature, left–as-made).

Amino Acid	C_p_ (%) [55]	C_s_ (%) [55]	C_p_ (%)	C_s_ (%)
Alanine	32.03	24.12	29.5±1.7	27.6±2.4
Arginine	0.10	1.31	0.5±0.1	0.4±0.4
Asparagine	0.00	0.00	1.3±0.7	1.8±1.2
Aspartic acid	0.42	4.53	0.9±0.4	1.3±0.8
Cysteine	-	-	0.0±0.1	0.0±0.0
Glutamic acid	0.34	3.12	0.4±0.3	0.4±0.1
Glutamine	0	0	1.1±0.6	2.2±1.1
Glycine	49.13	36.50	43.7±2.5	40.7±3.8
Histidine	0.04	0.54	0.4±0.3	0.7±0.2
Isoleucine	0.19	1.61	1.4±0.6	1.9±0.9
Leucine	0.10	1.71	1.3±1.0	2.5±1.4
Lysine	0.12	0.95	0.2±0.1	0.4±0.2
Methionine	0.03	0.31	0.1±0.1	0.4±0.2
Phenylalanine	0.34	1.47	0.5±0.0	0.6±0.1
Proline	0.13	0.96	0.1±0.0	0.2±0.1
Serine	11.79	9.20	10.8±1.6	10.2±0.4
Threonine	0.47	1.91	1.6±0.5	1.7±0.4
Tryptophan	0.00	0.00	0.0±0.1	0.0±0.1
Tyrosine	3.66	7.55	4.5±0.8	4.7±0.7
Valine	1.11	4.21	1.6±0.5	2.4±0.5

Regarding the charge of the FDPs (see Table 3), tabulating the LCMS results above (see Table 3) shows that overall the FDP is composed of neutral amino acids (97.6% and 96.8% for C_p_ and C_s_, respectively). Compared to the charge calculated from the composition of the FDPs obtained from the literature [55], where 7.6% of the amino acids are negatively charged, there is a significant difference in the charge of C_s_, as only 1.5% of the amino acids within the samples examined are negatively charged. It is unlikely that the C_s_ fragments incorporated into the hydrogel are attracted to the collagen, which is cationic in nature [27], as was previously assumed.

**Table 3:** Comparison of fibroin-derived polypeptides charge (right–from literature, left–as-made).

	Polypeptides charge			
	C_p_ (%) [55]	C_s_ (%) [55]	C_p_ (%)	C_s_ (%)
**positive**	0.22	2.25	1.3% ± 0.5%	1.7% ± 0.9%
**negative**	0.76	7.65	1.1% ± 0.5%	1.5% ± 0.8%
**neutral**	98.98	89.56	97.6% ± 0.9%	96.8% ± 1.7%

The SBF that was used to imitate a physiological environment for *in vitro* testing was analysed using Ion Chromatography. The results were compared to the theoretical composition of the ionic species present in solution as well as a commercial SBF solution (Hank’s Balanced Salt Solution, HBSS [67]) and Extracellular Fluid (ECF), specifically plasma [68]. The carbonate present in the SBF could not be measured due to on-column neutralisation of CO_3_^2-^ to HCO^3-^ by dissolved CO_2_.

**Fig 4:**
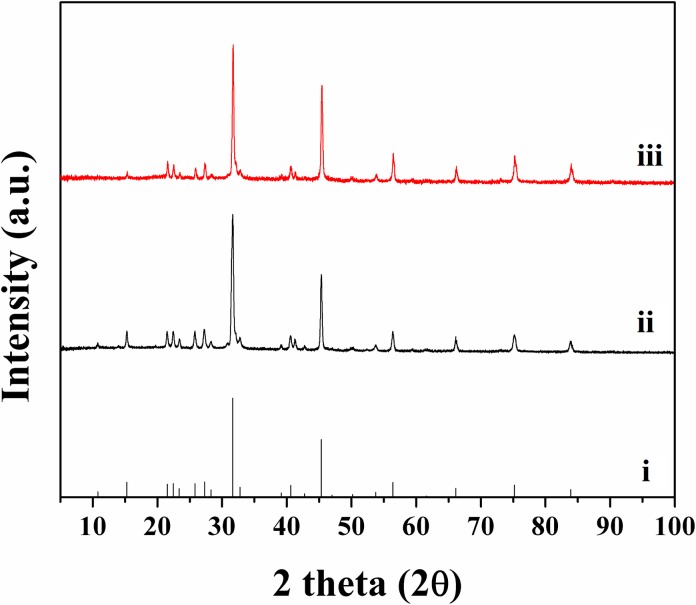
XRD patterns of i) HA (ICDD file 00-009-0432) and polypeptides ii) C_s_ and iii) C_p_ immersed in SBF.

Polypeptides (C_s_ and C_p_) immersed in SBF at 37 ºC showed aggregation within a day. The HA aggregate was confirmed from XRD analysis (see Fig 4). While HA can exist in different forms, depending on the condition of its formation [69], the method of nucleation remains the same. It is known that HA forms an intermediary amorphous calcium phosphate, that HA crystals exhibit “polycrystalline character of the elemental particles,” and nucleation is favoured [70], which would indicate that HA forms are determined by nucleation conditions.

The resulting XRD pattern showed peaks that matched the corresponding pattern for HA (ICDD file 00-009-0432), indicating that both polypeptides lead to the nucleation of HA at physiological conditions. Given the similar composition of C_s_ and C_p_ (see Table 2), it is to be expected that both would nucleate HA. Previous work has shown that the presence of acid-rich proteins (specifically aspartic and glutamic acid) led to biomineralization *in vitro* [71,72] via epitactic nucleation, whereas heterogeneous nucleation occurs via the formation of critical nuclei on a surface [73]. The XRD measurements of C_s_ and C_p_ immersed in SBF results in a diffraction pattern identical to that arising from HA.

### Hydroxyapatite nucleation investigation

Hydrogels incorporating C_s_ and C_p_ at a 1:10 additive to collagen ratio (by mass) were created as stated. SEM imaging was performed as described above. The results for DC, 1:10 DC-C_p_ and 1:10 DC-C_s_ gels, after seven days immersion in Kokubo’s SBF were analysed to visually determine whether HA nucleation occurred.

**Fig 5:**
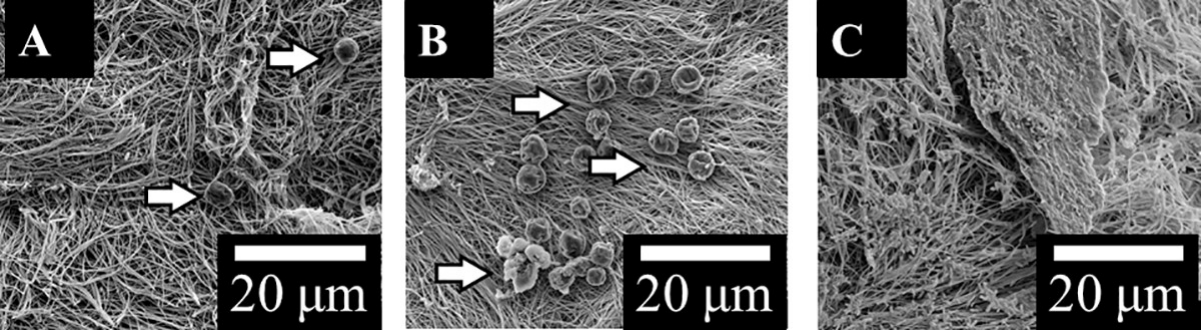
SEM of Plastically compressed A) DC, B) 1:10 DC-C_s_ and C) 1:10 DC-C_p_ hydrogels immersed in SBF for 7 days (scale bar is 10 μm, arrows indicate particles).

SEM reveals no major difference in the morphology of the as-made hydrogels, either DC, 1:10 DC-C_s_ or 1:10 DC-C_p_ (see Fig 5.). Particles that differed were seen in the DC and 1:10 DC-C_p_ gels at day 7, though there were no signs of particle nucleation in the 1:10 DC-C_p_ gels. In the DC/1:10 DC-C_s_ gels, the particles were located in small clumps of 1–20 particles randomly spaced across the surface of the hydrogel. Most of the hydrogel surface remained bare. No evidence of C_s_ is observed in the hydrogels via SEM at day 7, indicating that electrostatic interactions between collagen and the hydrophilic FDP [27] do not last.

**Fig 6:**
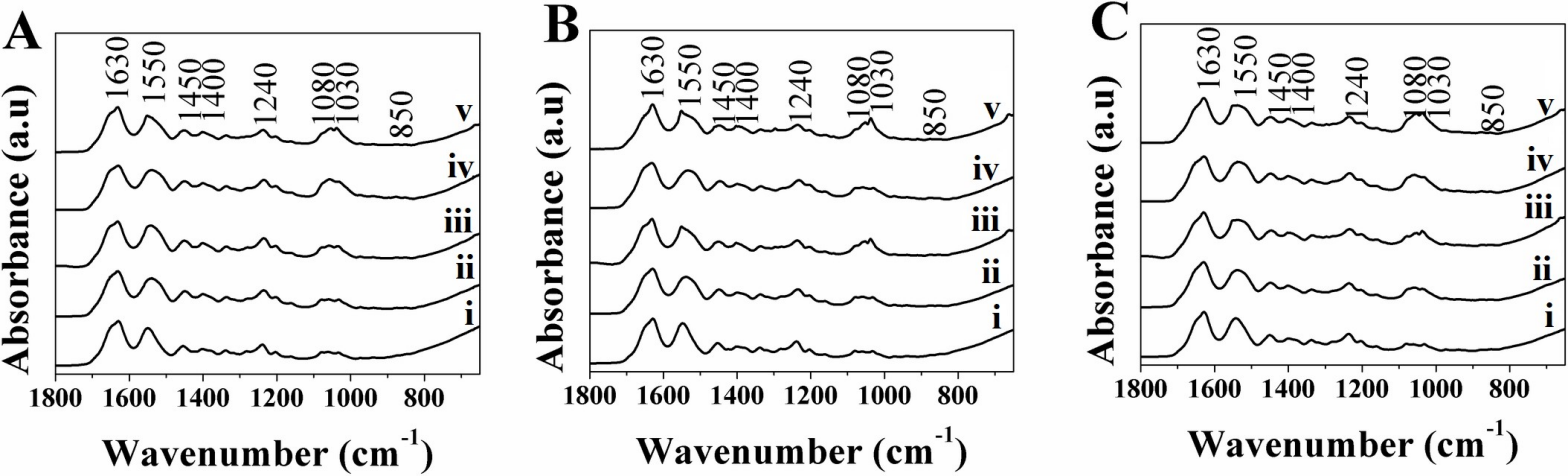
ATR-FTIR Spectra for A) DC, B) 1:10 DC-C_s_ and C) 1:10 DC-C_p_, gels immersed in SBF at day i) 0, ii) 3, c) 7, iii) 10 and iv) 14.

Spectroscopic and XRD analysis was conducted on DC hydrogels, both with and without additives incorporated, as described above. The resulting ATR-FTIR spectra were plotted and showed that bands indicative of the presence of HA; υ_3_ PO_4_^3-^ (1030 and 1080 cm^-1^) [58,74,75], as well as the bands for υ_3_ and υ_2_ CO_3_^2-^ (1450 and 1400 cm^-1^ and 850 cm^-1^ respectively) [58,74] were present in all samples, post-immersion in SBF. The presence of type I collagen is confirmed from the bands at 1630, 1550 and 1240 cm^-1^, corresponding to the amide I, II and II groups respectively [58,76] (see Fig 6) present in all samples.

The spectra obtained show that there was an increase in the υ_3_ PO_4_^3-^ over the first 10 days compared to the initial band, indicating the occurrence of HA nucleation/growth. This was particularly apparent in DC and 1:10 DC-C_s_ gels (see Fig 6A and 6B), as well as in the 1:10 DC-C_p_ gels (see Fig 6C).

**Fig 7:**
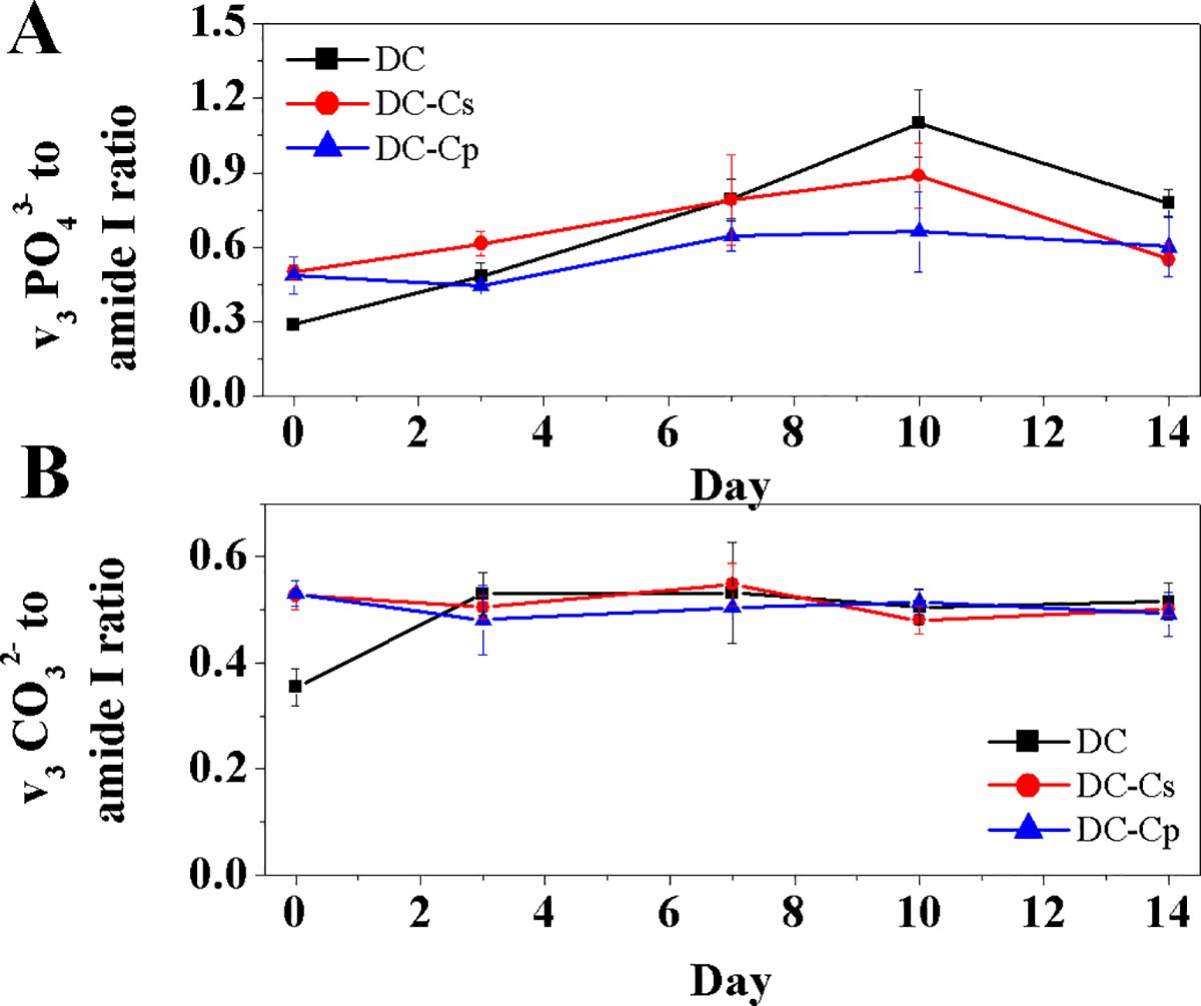
Ratio of A) ν3 PO_4_^3-^ and B) ν3 CO_3_^2-^ bands to amide I bands from ATR-FTIR spectra of DC gels containing no additives, C_s_ and C_p_ (at a 1:10 ratio to collagen) immersed in SBF (SD, n = 4, p<0.05).

The spectra indicated that all hydrogels showed a presence of phosphate and carbonate groups, suggesting some HA nucleation occurs within the hydrogel. Comparing the value of the peaks associated with phosphate and carbonate showed the bands associated with phosphate increased with time (see Fig 7A), those associated with carbonate did not (see Fig 7B), indicating that phosphate absorbance within the collagen increases over time. This change can be attributed to the mineralization of non-carbonated HA, and is consistent with previous findings [58]. The hydrogels that had C_s_ incorporated did not show significantly higher values associated with the phosphate peak, contrary to earlier work [27], indicating that it does not serve to nucleate HA at a higher rate. The ratio of phosphate and carbonate peaks to the Amide I band remained nearly constant over time, indicating that little change takes place within the 1:10 DC-C_p_ or C_s_ gels. It is likely that the FDPs distributed within the hydrogel lack the acidic amino acids, particularly glutamic acid, necessary for the nucleation of HA [71,72,77]. Additionally, the lack of electrostatic interaction between collagen and the mainly neutral FDP is also a barrier to HA formation, as it was shown to be necessary for mineralization to occur [73,78].

**Fig 8:**
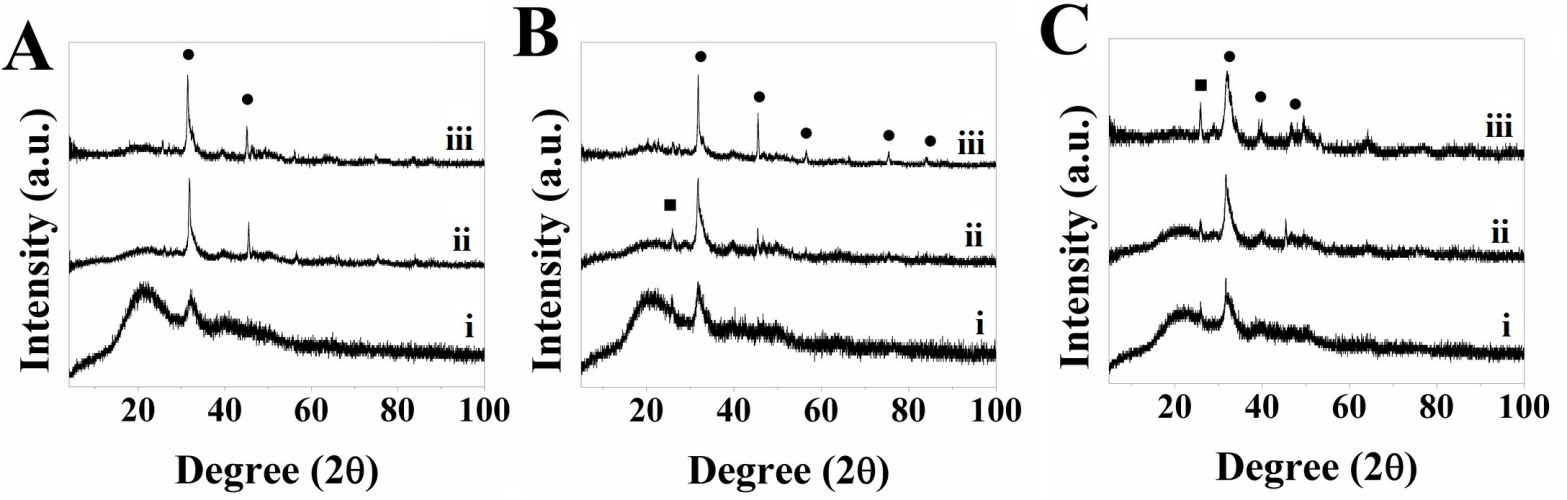
XRD pattern for A) DC, B) 1:10 DC-C_s_ and C) 1:10 DC-C_p_ hydrogels immersed in SBF at days i) 3, ii) 7, iii) 14 (●–HA corresponding to ICDD file 00-009-0432, ■–PE corresponding to ICDD file 00-060-1505).

The XRD patterns for DC hydrogels immersed in SBF showed that the formation of HA occurs in all samples and increases over time, though the extent of mineralization differs based on whether it was a DC, DC-C_p_ or DC-C_s_ hydrogel (see Fig 8). XRD patterns for pure collagen hydrogels showed peaks at 2θ = 33° and 45° in all samples, which are associated with HA [79]. The latter also exhibit a peak at 2θ = 23° in an otherwise amorphous section of the pattern. As collagen is an amorphous material with no defined peaks, and the 2θ = 23° peak does not match any peaks associated with HA, the presence of this peak has been attributed to the polyethylene (PE) tape used to hold the samples in place. In addition, the peak corresponds to ICDD file 00-060-1505 for PE.

XRD patterns confirmed the nucleation of HA in all samples. The 1:10 DC-C_s_ hydrogels (see Fig 8B) initially showed a similar extent of mineralization as DC and 1:10 DC-C_p_ gels on days 3 and 7. However, the XRD patterns for DC and DC-C_s_ hydrogels (see Fig 8A and 8B) showed much higher HA peaks at day 14, indicating a greater extent of mineralization than DC-C_p_ (see Fig 8C) hydrogels in the long-term. In particular, the peak at 2θ = 45° was not as apparent as in other DC and DC-C_s_ hydrogels. In general, however, both 1:10 DC-C_s_ and DC-C_p_ hydrogels showed that their incorporation led to HA nucleation, which can be explained by the fact that both C_s_ and C_p_ have similar amino acid compositions, as seen by the LCMS results, and the fact that both C_s_ and C_p_ lead to the nucleation of HA, as confirmed by the XRD pattern from the particles were immersed in SBF (see Fig 4). The major difference between C_s_ and C_p_ is related to the high, narrow peak at 2θ = 23°. The 2θ = 23° peak is present in all samples, but the intensity is much greater in the DC and DC-C_s_ samples. The broad peaks in the DC-C_p_ samples are attributed to a relatively small crystal size [80–82] or to the poorly crystalline nature [83,84] of HA within the sample. The XRD results match ATR-FTIR results, which shows that FDPs do not have a significant impact on HA nucleation due to the lack of the necessary acidic amino acids [71,72,77] or of the electrostatic interaction between collagen and FDP [73,78].

**Fig 9:**
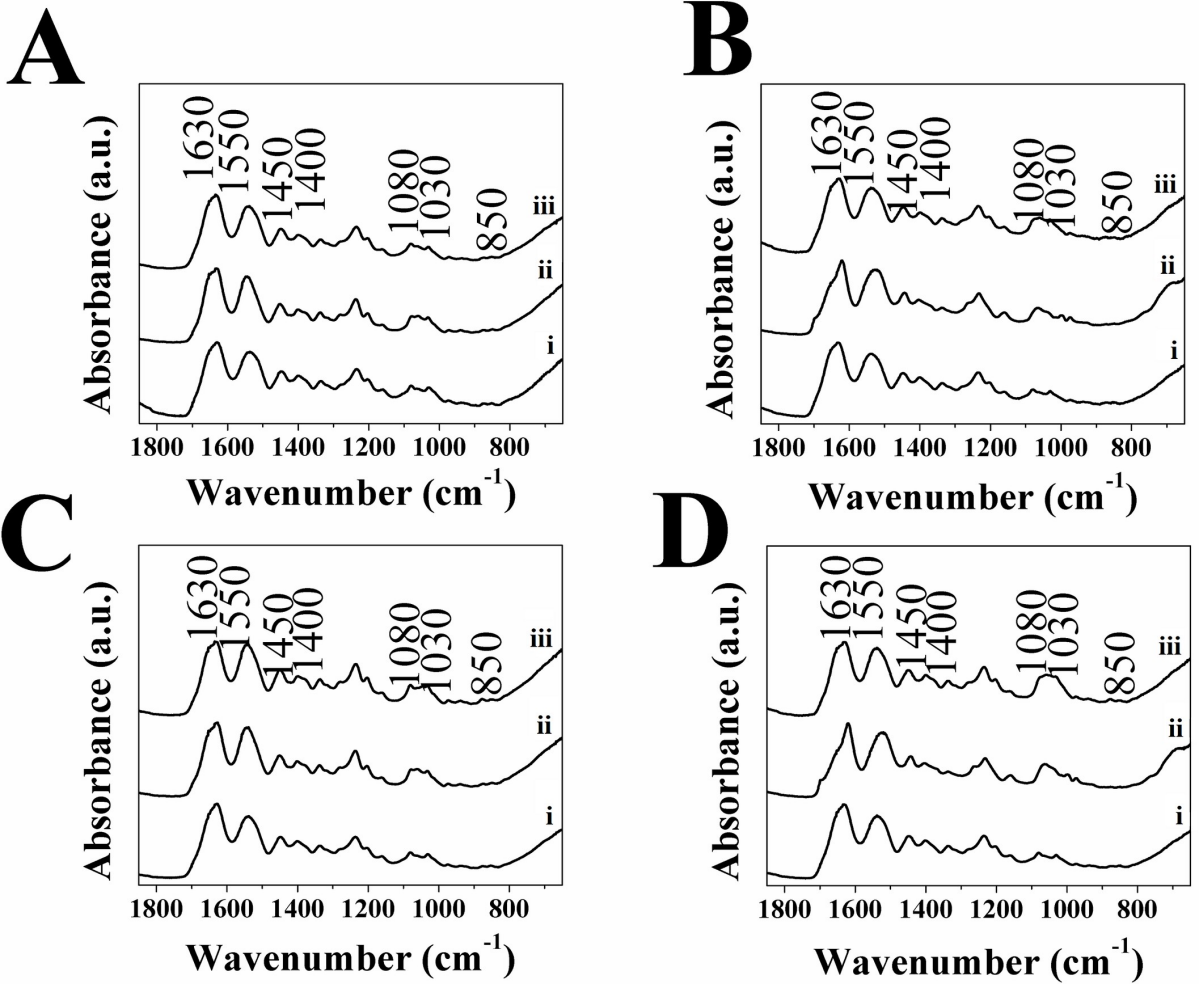
ATR-FTIR for A) 1:1 DC-C_s_, B) 1:1 DC-C_p_, C) 1:2 DC-C_s_ and D) 1:2 DC-C_p_, i) as-made and immersed in SBF for ii) 3 and iii) 7 days.

ATR-FTIR was also conducted on hydrogels with a higher amount of FDPs additive added (1:2 and 1:1) that were immersed in SBF for 3 and 7 days (see Fig 9). The resulting spectra for the different types of hydrogels, only DC-C_p_ showed that bands attributed to υ_3_ PO_4_^3-^ (1030 and 1080 cm^-1^) [58,74,75], increased with time, while the bands for υ_3_ and υ_2_ CO_3_^2-^ (1450 and 1400 cm^-1^ and 850 cm^-1^ respectively) [58,74] remained relatively constant, similar to the results obtained for additives added at a 1:10 ratio. The results obtained are contrary to the expectation that the inclusion of a greater amount of FPDs would lead to previously reported increased HA nucleation [27].

The mass of freeze-dried DC hydrogels with different ratios of C_s_ and C_p_ (1:2 and 1:1 relative to collagen) before and after immersion in SBF was measured to determine if the addition of FDP led to a change in mass due to HA nucleation over time.

**Fig 10:**
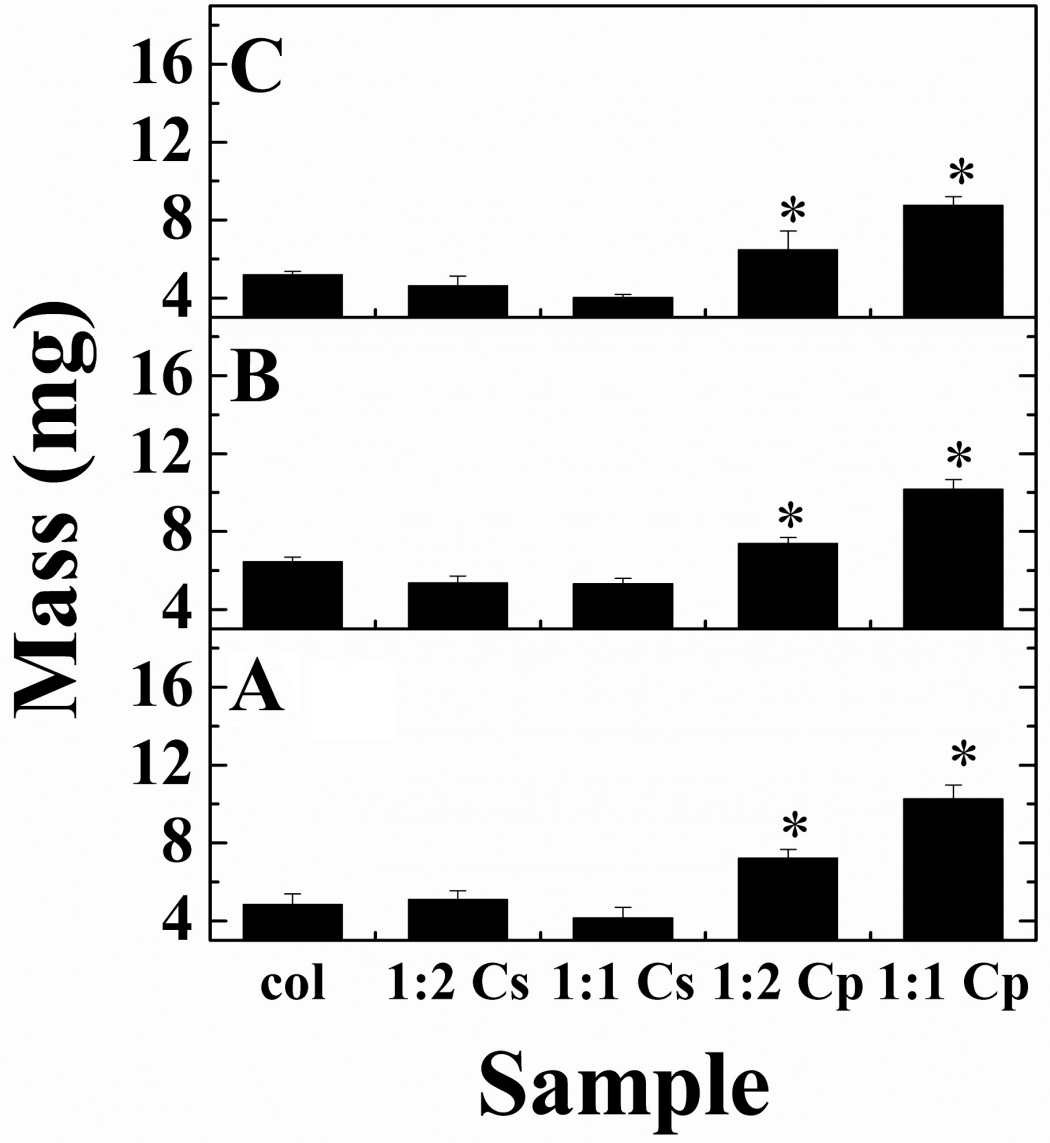
Mass of DC, DC-C_s_ and DC-C_p_ hydrogels immersed in SBF for A) 0 (as-made), B) 3 and C) 7 days (*—statistically significant, one-way ANOVA, SD, n = 4, p < 0.05).

Previously [27], it was reported that DC and DC-C_s_ hydrogels with 1:10 C_s_ to collagen should result in a hydrogel that is 5 wt.% and ~60 wt.% HA, respectively, by day 7 after immersion in SBF. However, the present results obtained show that there is no significant difference between plastically compressed DC gels and DC gels with C_s_ incorporated (see Fig 10). Comparison of the difference in the masses of the DC, DC-C_s_ and DC-C_p_ hydrogels to the theoretical value of an as-made (day 0) gel with the same amount of additive incorporated is reported in Table 4. The mass analysis of the DC gels after PC matched up closely (-3.6%) with the theoretical mass of a gel of the same volume. Results demonstrated that the DC-C_s_ hydrogels have a significantly lower mass than their theoretical value, while the mass of the DC-C_p_ gels is close to its theoretical value. Furthermore, the difference of mass in the DC-C_s_ was similar to the amount of C_s_ added to the solution, and statistically, the difference in mass between the DC and DC-C_s_ gels is not significant. This indicates that C_s_ is expelled with the water inside the gel during the plastic compression of the highly hydrated hydrogel, and that the remaining scaffold is only collagen.

**Table 4:** Mass of hydrogels at day 0 compared to the theoretical values.

Sample	Theoretical mass (mg)	Initial mass (mg)	Difference in mass (%)	Difference in mass from control (mg)
DC (ctrl)	4.7	4.9±0.5	3.6	0.0
1:2 DC-C_s_	7.0	5.1±0.5	-27.4	0.2
1:1 DC-C_s_	9.4	4.2±0.5	-55.7	-0.7
1:2 DC-C_p_	7.0	7.2±0.4	3.0	2.3
1:1 DC-C_p_	9.4	10.3±0.7	9.7	5.4

### Conclusions and perspectives

We examined the interaction *in vitro* between silk fibroin-derived polypeptides (FDPs) and plastically compressed collagen hydrogels developed as scaffolds for bone tissue engineering. Results *in vitro* show that immersing FDPs (both C_s_ and C_p_) in simulated body fluids lead to the nucleation of hydroxyapatite (HA), yet incorporating these same FDPs within a dense collagen (DC) hydrogel does not contribute to mineralizing the collagen, as was previously reported [27]. Characterization of the polypeptides using Liquid Chromatography–Mass Spectrometry showed the presence of glutamic acid which is important to promoting HA nucleation within the protein. Glutamic acid was present in a lower quantity than expected, especially for C_s_, though this may be attributed to the different experimental techniques used to quantify the amino acid composition or its processing. The results indicate that it is less suitable for promoting biomineralization than was initially thought, but it is possible that this may vary depending on the source and processing of the silk. Furthermore, mass analysis of DC hydrogels containing C_s_ (DC-C_s_) over time indicated that the C_s_ fragments are immediately expelled during plastic compression (PC). The silk fibroin-derived polypeptides incorporated into a collagen hydrogel show little difference compared to a pure collagen hydrogel for promoting the nucleation of hydroxyapatite, and it cannot serve as a replacement for non-collagenous proteins (NCPs) in bone tissue engineering (BTE) without first refining the method of linking the polypeptide to the collagen fibril.

## Supporting information

S1 File ATR-FTIR dataset for silk FDPs. (XLSX)

S2 File XRD dataset for silk FDPs. (XLSX)

S3 File PSA dataset for C_s_ and C_p_. (XLSX)

S4 File Raw data from LCMS for silk FDPs. (XLSX)

S5 File XRD dataset of C_s_ and C_p_ in SBF. (XLSX)

S6 File ATR-FTIR dataset for DC, DC-C_s_ and DC-C_p_ hydrogels immersed in SBF for 0–14 days. (XLSX)

S7 File Analysis of dataset of ATR-FTIR for DC, DC-C_s_ and DC-C_p_ hydrogels immersed in SBF for 0–14 days. (XLSX)

S8 File XRD dataset of ATR-FTIR for DC, DC-C_s_ and DC-C_p_ hydrogels immersed in SBF. (XLSX)

S9 File ATR-FTIR dataset for 1:1 and 1:2 DC-C_s_/DC-C_p_ hydrogels, as-made and immersed in SBF for 3 and 7 days. (XLSX)

S10 File Recorded masses of DC, 1:2 DC-C_s_, 1:1 DC-C_s_, 1:2 DC-C_p_, and 1:1 DC-C_p_ hydrogels after immersion in SBF for 0, 3 and 7 days, and statistical analysis of the results. (XLSX)

S11 File Values for the concentration of ions in ECF, HBSS and Ion Chromatography results for SBF. (XLSX)

S12 File ATR-FTIR dataset for bone, as-received and after heat treatment. (XLSX)

S13 File XRD dataset for bone, as-received and after heat treatment. (XLSX)

S1 Fig Schematic of method used for plastic compression The schematic shows the plastic compression of a collagen hydrogel based on the protocol established by Brown et al. [33]. An uncompressed (highly hydrated) hydrogel is placed between two pieces of nylon mesh, and placed overtop a steel mesh and a paper towel (blot paper). A weight (glass plate) is placed on top the hydrogel to produce a constant force (1 kPa) and held for 5 minutes. Water is expelled from the bottom, through the nylon and steel meshes, and soaked up by the blot paper. The hydrogel is plastically compressed into a dense collagen hydrogel, and then is removed from between the nylon meshes.
(TIF)

S2 Fig ATR-FTIR spectra for bone (bovine) i) as-received and i) after heat treatment. FTIR spectra shows that bovine bone has the characteristic peaks of collagen from the bands amide I, II and II groups at 1630, 1550 and 1240 cm^-1^ [58,76], while the presence of HA is seen in the large phosphate band at 1030 and 1080 cm^-1^ [58,74,75]. The results are supported by those seen in literature [85–87] of FTIR conducted on bone and bone samples that have had collagen removed.
(TIF)

S3 Fig XRD spectra for bone (bovine) i) as-received and i) after heat treatment. **iii) ICDD file 00-046-0905.** XRD analysis show that the samples of bone match that seen in literature [88,89]. Heat treatment reveals the band pattern of bone and calcined bone, with the peaks of the latter matching that of calcium-deficient hydroxyapatite (CDHA) (ICDD file 00-046-0905).
(TIF)

S4 Fig Analysis of Kokubo’s SBF via IC compared to theoretical values for Kokubo’s SBF and HBSS, as well ECF (plasma). Ion Chromatography of Kokubo’s SBF shows that the composition is nearly identical to the calculated theoretical values, as well as the composition of commercial SBF (Hank’s Balance Salt Solution, HBSS) [67]. In addition, it is also similar to the composition of ECF [42], with the exception of the amount carbonate, which is taken to be much lower than that of ECF given the theoretical value.
(TIF)
