# Supplementary figures and images for: Silk fibroin-derived polypeptides additives to promote hydroxyapatite nucleation in dense collagen hydrogels
Source: PLoS One. 2019 Jul 15;14(7):e0219429. doi: 10.1371/journal.pone.0219429 (PMC6629059; doi:10.1371/journal.pone.0219429)

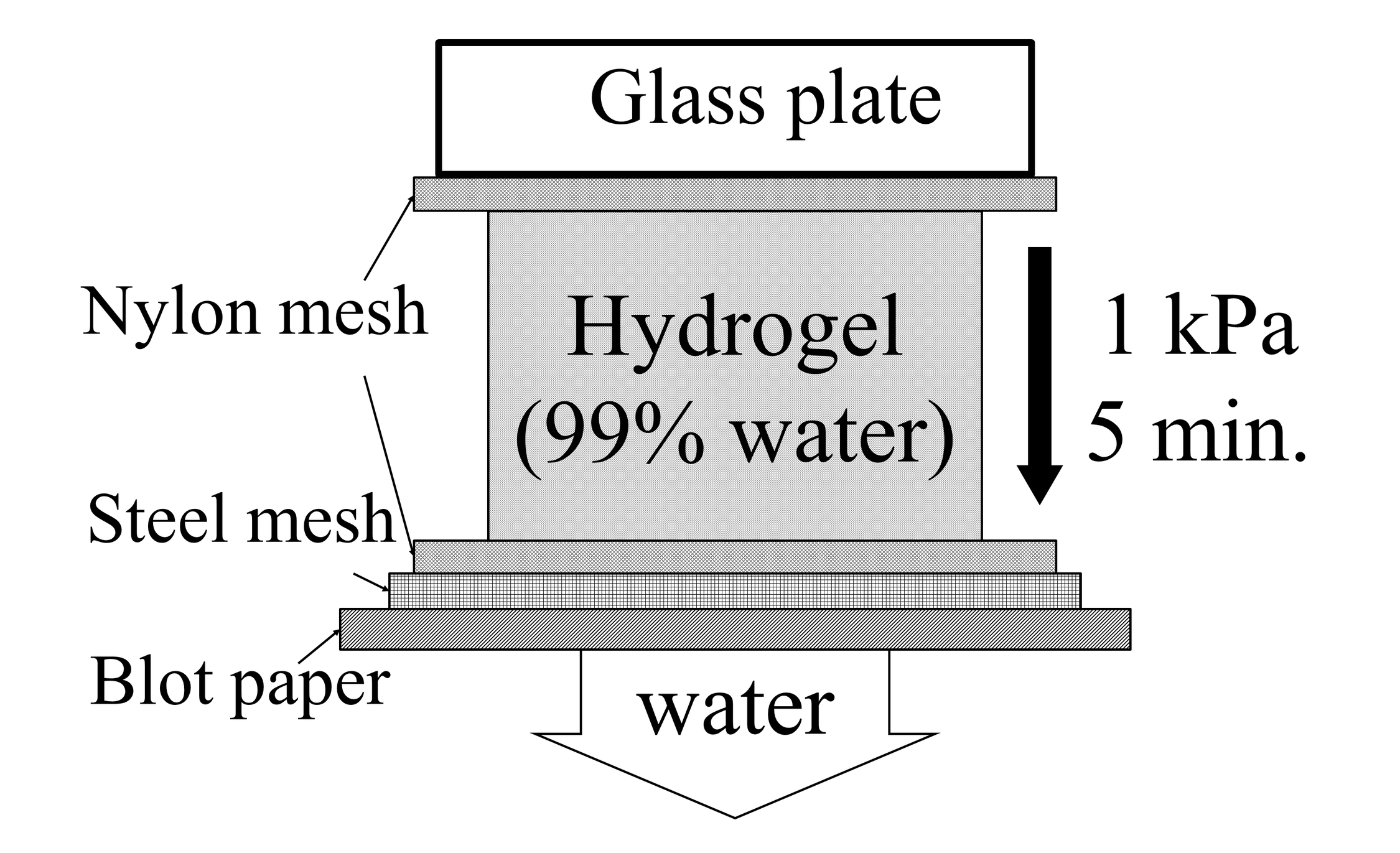

Supplement: S1 Fig — An uncompressed (highly hydrated) hydrogel is placed between two pieces of nylon mesh, and placed overtop a steel mesh and a paper towel (blot paper). A weight (glass plate) is placed on top the hydrogel to produce a constant force (1 kPa) and held for 5 minutes. Water is expelled from the bottom, through the nylon and steel meshes, and soaked up by the blot paper. The hydrogel is plastically compressed into a dense collagen hydrogel, and then is removed from between the nylon meshes. (TIF) [file pone.0219429.s014.tif]

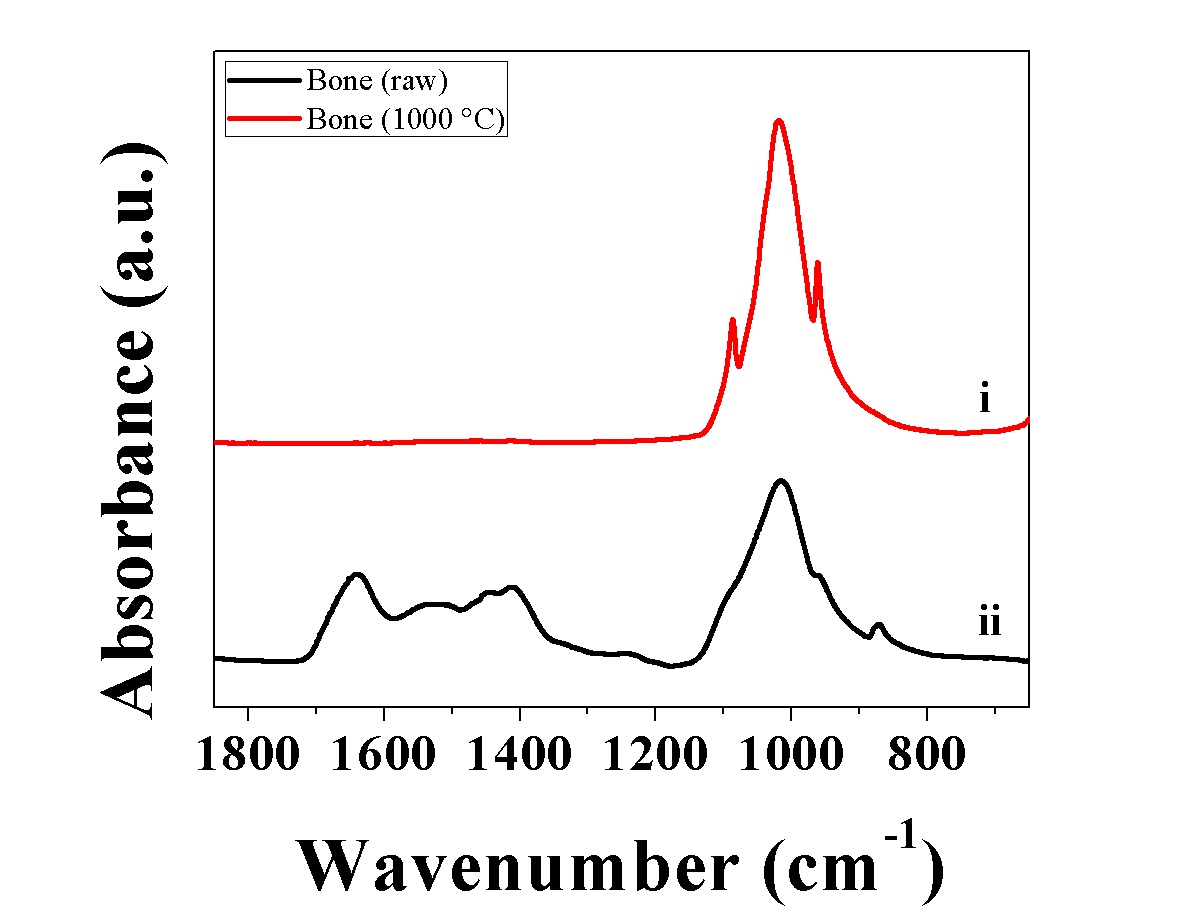

Supplement: S2 Fig — FTIR spectra shows that bovine bone has the characteristic peaks of collagen from the bands amide I, II and II groups at 1630, 1550 and 1240 cm-1 [58,76], while the presence of HA is seen in the large phosphate band at 1030 and 1080 cm-1 [58,74,75]. The results are supported by those seen in literature [85–87] of FTIR conducted on bone and bone samples that have had collagen removed. (TIF) [file pone.0219429.s015.tif]

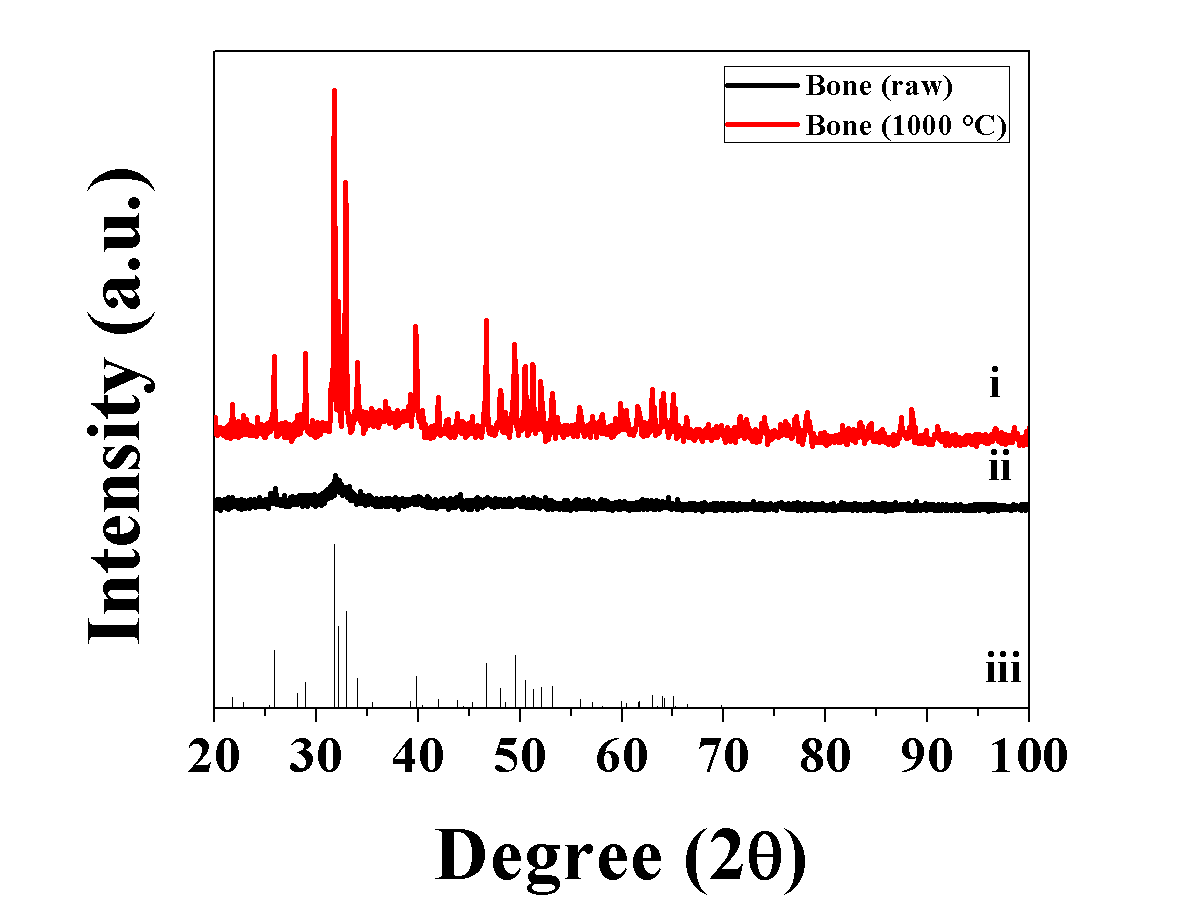

Supplement: S3 Fig — iii) ICDD file 00-046-0905. XRD analysis show that the samples of bone match that seen in literature [88,89]. Heat treatment reveals the band pattern of bone and calcined bone, with the peaks of the latter matching that of calcium-deficient hydroxyapatite (CDHA) (ICDD file 00-046-0905). (TIF) [file pone.0219429.s016.tif]

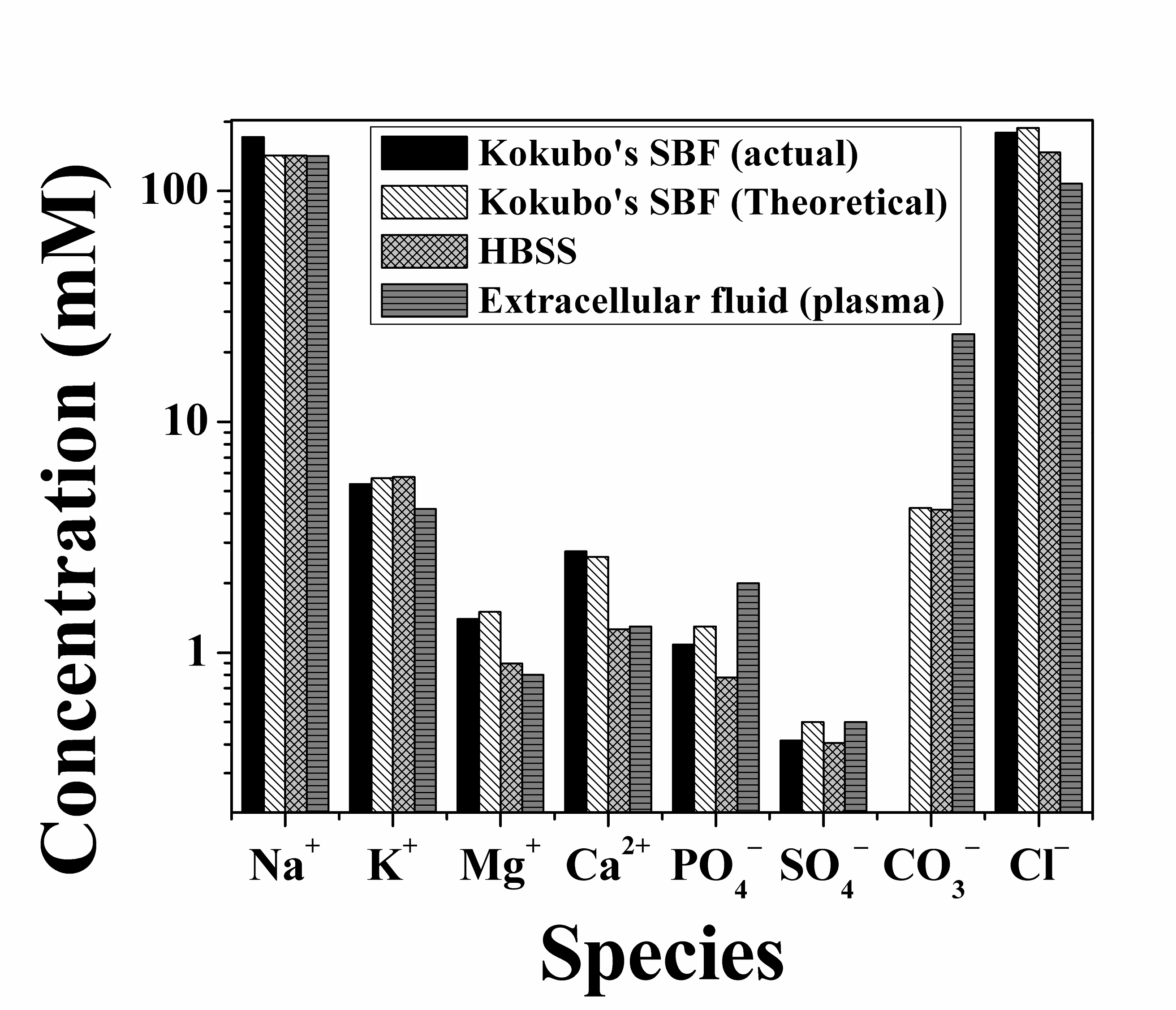

Supplement: S4 Fig — Ion Chromatography of Kokubo’s SBF shows that the composition is nearly identical to the calculated theoretical values, as well as the composition of commercial SBF (Hank’s Balance Salt Solution, HBSS) [67]. In addition, it is also similar to the composition of ECF [42], with the exception of the amount carbonate, which is taken to be much lower than that of ECF given the theoretical value. (TIF) [file pone.0219429.s017.tif]
